# The Role of Nanoanalytics in the Development of Organic-Inorganic Nanohybrids—Seeing Nanomaterials as They Are

**DOI:** 10.3390/nano9121673

**Published:** 2019-11-23

**Authors:** Daria Semenova, Yuliya E. Silina

**Affiliations:** 1 Process and Systems Engineering Center (PROSYS), Department of Chemical and Biochemical Engineering, Technical University of Denmark, 2800 Kgs. Lyngby, Denmark; dsem@kt.dtu.dk; 2 Institute of Biochemistry, Saarland University, 66123 Saarbrücken, Germany

**Keywords:** hybrids, organic-inorganic nanomaterials, nanoanalytics, characterization, LDI-MS platform, surface chemistry, nanobiosensors, standardization

## Abstract

The functional properties of organic-inorganic (O-I) hybrids can be easily tuned by combining system components and parameters, making this class of novel nanomaterials a crucial element in various application fields. Unfortunately, the manufacturing of organic-inorganic nanohybrids still suffers from mechanical instability and insufficient synthesis reproducibility. The control of the composition and structure of nanosurfaces themselves is a specific analytical challenge and plays an important role in the future reproducibility of hybrid nanomaterials surface properties and response. Therefore, appropriate and sufficient analytical methodologies and technical guidance for control of their synthesis, characterization and standardization of the final product quality at the nanoscale level should be established. In this review, we summarize and compare the analytical merit of the modern analytical methods, viz. Fourier transform infrared spectroscopy (FTIR), RAMAN spectroscopy, surface plasmon resonance (SPR) and several mass spectrometry (MS)-based techniques, that is, inductively coupled plasma mass spectrometry (ICP-MS), single particle ICP-MS (sp-ICP-MS), laser ablation coupled ICP-MS (LA-ICP-MS), time-of-flight secondary ion mass spectrometry (TOF-SIMS), liquid chromatography mass spectrometry (LC-MS) utilized for characterization of O-I nanohybrids. Special attention is given to laser desorption ionization mass spectrometry (LDI-MS) as a reliable nanoanalytical platform for characterization of O-I hybrid nanomaterials, their quality, design verification and validation.

## 1. Introduction

Analytical chemistry devoted to nano-sized objects has developed considerably over the last decade. It should be noted that the evolution in nanotechnology itself, to a substantial degree, occurred due to the development of corresponding analytical methods and strategies. Meanwhile, the term “nanoanalytics” is rarely used in the scientific literature and is mostly related to the field of surface, interface and thin-film micro-area analysis. A literature survey on the “nanoanalytics” term, using the Web of Science database (www.webofknowledge.com) identified only 21 documents (h-index 7) published from 2008 to 2019, clearly indicating that this is a relatively new research field. However, at the same time, the search performed for the term “characterization of nanomaterials” revealed more than 6500 documents (h-index 160) published during the same years. Meanwhile, “characterization of nanomaterials” can be readily considered as a more likely routine approach with some emphasis on instrument precision and personal hands-on skills as compared to “nanoanalytics” where the fundamental knowledge of methodology development and validation, physics, chemistry, biochemistry and nanoscience are requested.

Such an absurd situation is caused by the fact that analytical chemistry in general, and nanoanalytics as one of its units, are often considered by the major users (mainly inorganic chemists, material scientists, physicists and biologists) as a routine procedure rather than a fundamental scientific approach.

A recent statement by Prof. Y. Zolotov clearly summarizes the current state-of-the-art in analytical chemistry: “The opinion of some snobs in science that analytical chemistry is not an independent science and should only serve others, including ‘their’ fields of science is worth I-don’t-know-what. These snobs do not know the difference between analytical science and analytical service, as a system of analytical support of related and unrelated sciences’’ [1]. Furthermore, Karayannis and Eftathiou, addressing the role of modern analytical and nanoanalytical chemistry, claim that this is “the key to solve problems related to material systems and to society” [1].

Therefore, the global progress within hybrid nanomaterials applied for the development of lab-on-chip devices, miniaturized analytical systems (*µ*-TAS), microfluidic devices, nanoparticles-based bioassays, electrochemical biosensors, devices for clinical and environmental monitoring and so forth, would be absolutely impossible to imagine without solutions proposed by analytical chemistry [2,3,4,5].

Moreover, the mass production of nano-based systems revealed a wide spectrum of physical limitations of several characterization techniques because nano-phenomena appearing at small dimensions can be poorly controlled [6]. In this regard, certain descriptive functions of nano-objects and analytical methodologies related to the nano-characterization of such materials, the estimation of their traceability and validation strategies should be clearly defined.

Recently, Lespes et al. published an excellent review on instrumental nanoanalytical methods, principal concepts and specificities where the authors clearly defined the term “nano-object,” utilized in the methods and fields of their applications [6]. However, clear guidelines for the choice of nanoanalytical assays or platforms for the comprehensive characterization of nanomaterials remains a challenge.

Thus, it is believed that the role of analytical chemistry in the development and characterization of the materials, particularly for such complex systems as hybrid nanomaterials, must be re-considered and the basic principles towards their design validation and characterization must be highlighted.

Organic-inorganic (O-I) hybrids are a relatively novel class of nanomaterials that have found various applications in biomedical devices, as well as in bio- and environmental sensing tools [7]. This unique class of nanomaterials combines the beneficial features of both the organic and inorganic components. Moreover, the ability to tune the properties of hybrid nanomaterials through the combination of functional components opens up the possibilities for tailoring the surface chemistry depending on task requirements.

However, the manufacturing of O-I nanohybrids often suffers from mechanical instability leading to irreversible changes in device architecture and morphology that eventually results in insufficient signal reproducibility and degradation of the overall nano-based systems. Moreover, the synthesis reproducibility from batch-to-batch for such O-I hybrid nanomaterials is very low. On the other side, the properties of the final hybrid products are directly related to properties of the incorporated nano-components. Therefore, appropriate nanoanalytical methods and procedures for the control of synthesis reproducibility and quality characterization at the nano-scale of the final end-products have to be developed and validated.

A wide spectrum of analytical techniques was applied to validate the synthesis of the hybrid nanomaterials, viz. conventional microscopy-based techniques (scanning electron microscopy (SEM), transmission electron microscopy (TEM), scanning tunneling microscopy (STM), atomic force microscopy (AFM) etc.) and photoelectron spectroscopy (X-ray photoelectron spectroscopy (XPS), ultraviolet photoelectron spectroscopy (UPS), Auger electron spectroscopy (AES) etc.). However, the mentioned analytical methods do not allow detection of the minor changes in the O-I nanohybrids architecture and chemistry. Therefore, the development of tools and nanoanalytical approaches that could allow a deeper insight into the fabrication, design validation and characterization of the hybrid nanomaterials (i.e., O-I hybrids as a case study) in principle should significantly accelerate the progress in the field of material sciences, nanoscience, lab-on-chip, bio-and environmental sensing.

In this review we aim to (i) illustrate the conceptual strategies in nanoanalytics as a tandem of several assays which should be utilized in the development of the O-I hybrid nanomaterials; (ii) to summarize the methodologies used for characterization of O-I nano-objects with a general emphasis on laser desorption ionization mass spectrometry (LDI-MS) as a reliable nanoanalytical platform for metrology, validation of the hybrid nanomaterials and their standardization.

## 2. Organic-Inorganic Hybrids as a Targeted Class of Nanomaterials—From Classification to Mechanistic Aspects and Validation

**Figure 1:**
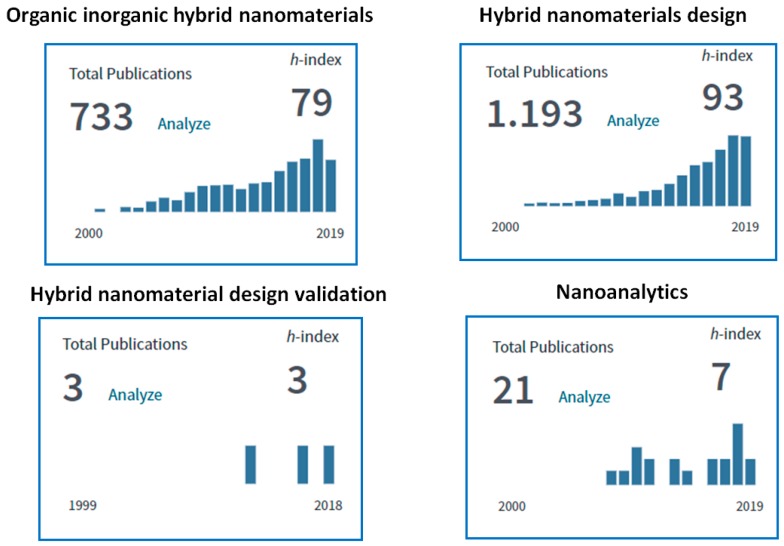
The number of cited papers related to the terms highlighted above per year. Preliminary figures for the present decades indicate a continuation of this trend. (Compiled from the Web of Knowledge database search, Oct. 2019), www.webofknowledge.com.

During the last two decades, organic-inorganic (O-I) hybrid nanomaterials have rapidly become a fascinating and incredibly rapidly developing new research field in materials science. Figure 1 (*top*) shows the importance and impact of the field in nanoscience and nanoengineering.

In general, O-I nanohybrids can be broadly defined as nanocomposites with intimately mixed organic (biological) and inorganic networks [8]. However, O-I hybrid nanomaterials are not simply physical mixtures but are more likely well-structured assemblies at the molecular scale, having at least one component, either the organic or inorganic component at the nanodimensions (≤100 nm). Remarkably, the final properties of O-I hybrid nanomaterials can (i) result from the sum of the individual intact compounds or (ii) from the strong synergy phenomenon created by the nanohybrid interface [9,10,11,12,13].

The architecture, design, stability, morphological and chemical properties of O-I hybrid nanomaterials are not only affected by synthesis conditions, that is, preparation method, pH, temperature, contact time and so forth, but also by varying the amount and ratio of the organic or inorganic networks. Therefore, O-I hybrid nanomaterials have a great potential towards the development of high-performance tailored materials that can be applied in various fields, viz. colloid templates, hollow capsules, functional nanogels, optics, micro-electronics, biohybrids, nanomembranes, on-demand release smart nanomaterials, stimuli responsive nanomaterials, nanobiosensors and so forth.

**Figure 2:**
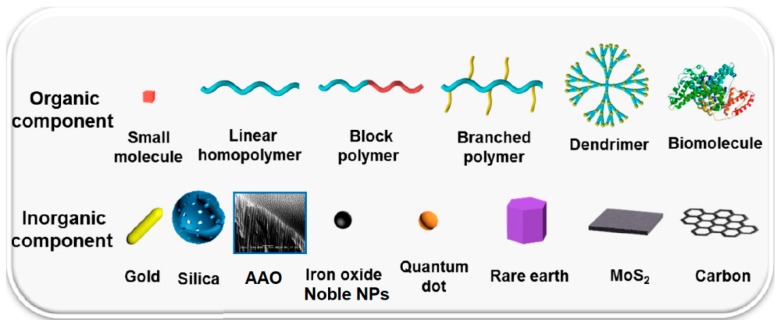
Typical organic and inorganic components employed in nanohybrids.Note: AAO—anodic aluminum oxide; Noble NPs—nanoparticles of noble metals. Reproduced and modified with permission of [14]. Copyright American Chemical Society, 2018.

In hybrid nanomaterials, an inorganic part plays several roles, that is, enhancing the mechanical and thermal stability, improving charge transfer and RedOx activity of the system, as well as providing specific magnetic, electronic, electrochemical or chemical properties. In contrast, organic components can contribute to specific physical or (bio)chemical properties of the final interfaces (i.e., electrical, electrochemical, specific RedOx potentials, optical characteristics, etc.). By means of the organic compound it is possible to enhance/modify the mechanical properties of surfaces, to control the porosity/density and attachment behavior of networks, to tailor hydrophilic/hydrophobic balance of the surface and control the porosity/density and attachment behavior of O-I networks. The examples of common organic and inorganic components employed for the synthesis of the nanohybrids are summarized in Figure 2.

The O-I nanohybrids can be divided in two classes [15]: **Class I**, organic and inorganic components are linked into networks by means of exchange weak bonds, viz. Van der Waals interactions, hydrogen bonds or ionic bonds formation. Namely this class of nanohybrids thanks to their hybrid chemistry has found applications in sensors, biosensors and catalysts development.

**Figure 3:**
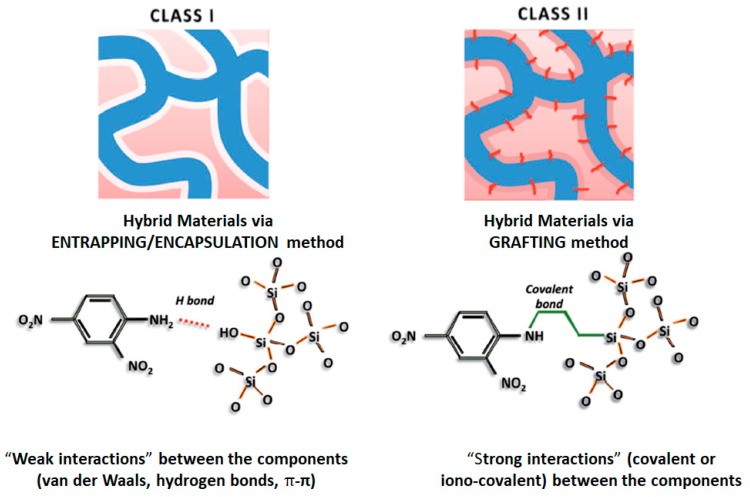
Classification of O-I hybrids. Reproduced and modified with permission of [19]. Copyright John Wiley and Sons, 2018.

In **Class II**, both O-I components are linked via covalent or iono-covalent bonding such as organo-mineral co-polymers [16,17,18]. The presence of covalent chemical bonds between organic and inorganic components exhibits several advantages which can be summarized as follows—synthesis of novel nanomaterials with improved functional properties, potentially enhanced formation of O-I interface and tailored hydrophilic-hydrophobic balance. Also, it should be taken into account that many hybrid nanomaterials may have both types of interactions at once which are typically seen in **Class I** or **Class II**, that is, weak Van der Waals and strong covalent interactions together, see Figure 3.

**Figure 4:**
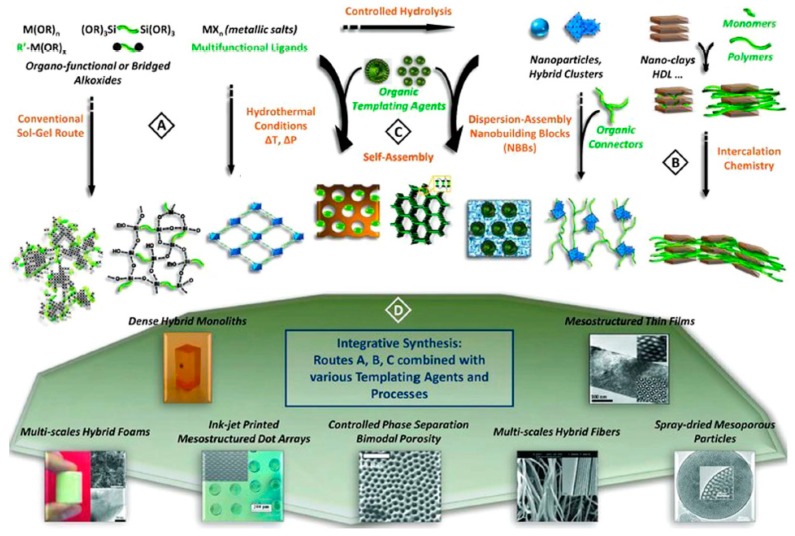
The summary of the available synthetic methodologies applied for the production of O-I nanohybrids. (A)–(D)—preparation routes. Reproduced with permission of [16]. Copyright John Wiley and Sons, 2010.

Regardless of the class of nanohybrids, they can be prepared in several ways (Figure 4)—by conventional sol-gel chemistry or by hydro- and solvothermal synthesis (Route A); via hybridization of well-defined nanobuilding blocks (NBBs)/assemblies of preformed monodispersed nano-objects (Route B); by self-assembly of amphiphilic molecules and polymers to generate supramolecular templates and to control the texture and morphology of the growing solid or gel phase (Route C) and by means of several tandem methods (Route D).

The conventional sol-gel pathway allows for simple and low-cost hybrid networks which are obtained through hydrolysis of organically modified metal alkoxides (Route A). The Route B is usually used to obtain nanohybrids with a better definition of inorganic component. In order to form interconnected hybrid networks, the NBBs might be functionalized via polymerization or connected by organic ligands. The Route C leads to the formation of self-assembled hybrids with a controlled texture and morphology.

**Figure 5:**
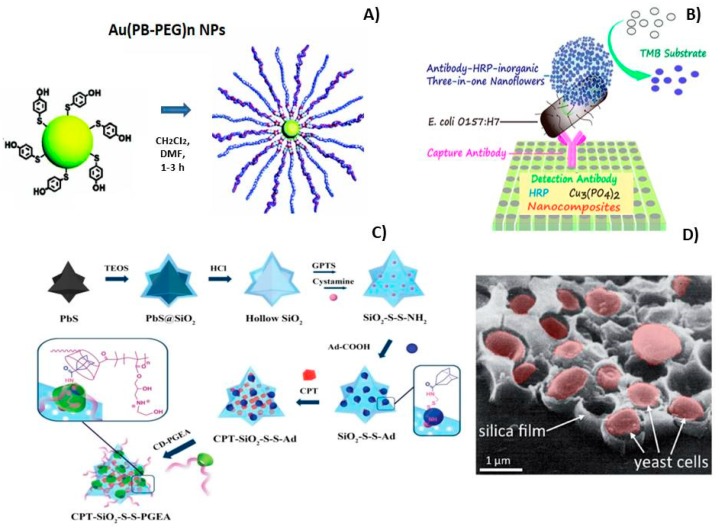
Some examples of functionalized O-I nanohybrids: (**A**) Au-NPs on the films of polythiophene derivatives through a covalent bond formed between the dithiolane moiety and gold; (**B**) Schematic representation of the Hydrangea-Like Antibody-Enzyme-Inorganic Three-in-One Nanocomposite Based Ultrasensitive Enzyme-Linked Immunosorbent Assay (ELISA) for Escherichia coli detection. (**C**) Schematic illustration of the self-assembly strategy for preparation of star-like nanohybrids through CD-Ad (cyclodextrin-adamantane) by host-guest interaction. Note: CD-PGEA- gene vector consisting of β-CD cores and ethanolamine (EA)-functionalized poly (glycidyl methacrylate). (**D**) Silica coatings containing immobilized living yeast (*Saccharomyces cerevisiae*). Reproduced and adapted with permission of [25,26,27,28,29]. Copyright Elsevier, 2005, 1993; American Chemical Society, 2016; The Electrochemical Society, 2019; John Wiley and Sons, 2015.

The fabrication strategies of advanced O-I hybrid nanomaterials were highlighted in several critical and tutorial reviews [18,19]. The examples of successful fabrication of O-I nanohybrids through the described above synthesis procedures are summarized in Figure 5. Thus, the formation of metal-sulfur bond is often used for grafting polymers with thiol groups onto metallic carries. The most well-known example is Au-thiol bonding which constitutes the predominant interaction of Au-NPs with thiolated polymers (Figure 5A) [20,21,22,23,24,25]. Another example includes the formation of protein-inorganic hybrid nanoflowers for Enzyme-Linked Immunosorbent Assay (ELISA) using hydrangea-like antibody-enzyme-inorganic nanocomposites, (Figure 5B) [26]. Interesting results were obtained via self-assembly strategy for nanohybrids preparation by host-guest interaction, Figure 5C. The final supramolecular O-I nanohybrid structure included an encapsulated anticancer drug (10-hydroxy camptothecine, CPT) in the hollow of the cavities of the bioreducible host-guest system. This design guarantees the release of the drug in a controlled manner. Beyond ELISA-like assays or host-guest systems, a great advancement in the field consisted of the entrapping of biomolecules or living cells into the inorganic networks, Figure 5D [27].

However, it should be mentioned that produced nanohybrids are usually amorphous with polydispersed structures, which makes it difficult to study their fundamental structure-properties relationships. Moreover, within O-I nanohybrids, a wide range of possible architectures can be distinguished starting from sandwich-like structures, cellular, lattice structured assemblies to segmented structures, porous materials and more. The number of possible combinations of organic or inorganic networks, matrices, fillers, additives and configurations opens up the possibilities for design modifications with O-I nanohybrids for further properties’ improvement and tailoring.

In 2010, Ashby et al. proposed the “Hybrid Synthesizer“—a theoretical tool that allows predicting and scanning the properties of novel hybrids by combining and comparing the properties of the incorporated monolithic materials [30]. This tool applies continuum and micro-mechanical models to estimate the equivalent properties of certain configurations seeking the best ones that meet a given set of design requirements. In addition, this platform allows approximation of the analysis of properties of the virtual hybrids that might be achieved by forming a single material into cellular or sandwich-like structures. However, it should be mentioned that “Hybrid Synthesizer” can properly predict the properties of the hybrid materials only via fully validated and experimentally obtained configurations [30]. Thus, the optimization or development of the crucial analytical tools for hybrid nanomaterials design screening and validation in general, and O-I nanohybrids as a case study, would be a desirable alternative.

Once a validation platform is developed, it can significantly encourage the innovations in virtual nanohybrids design by allowing formation of the comprehensive database of the appropriate O-I nanohybrids and their properties. Currently, the validation methodology of the hybrid nanomaterials seems to be under development (see Figure 1 (bottom)).

In addition, it should be noted that one of the greatest problems in nanomaterials science is the absence of generally accepted methods and methodologies for the characterization of an increasing number of complex nano-objects, that is, nanohybrids. In this case, the lack of both reproducibility and quality control tests appears to be a serious problem that makes the fabrication of the nanohybrids with guaranteed properties and behavior almost impossible.

Remarkably, the validation and metrology fields belong to the expertise area of analytical chemistry. At the same time, analytical chemistry takes advantage of the new possibilities and phenomena offered by nanoscience and nanotechnology and applies it to developing new methods for the analysis and characterization of nanohybrids. In this regard, the new branch of analytical chemistry of so-called “nanoanalytics” was recently introduced [31].

The next part of this review will be focused on tools and strategies utilized in nanoanalytics for the analysis and validation of nanomaterials in general and O-I nanohybrids as a case study.

## 3. Nanoanalytics: Seeing Nanomaterials as They Are

**Figure 6:**
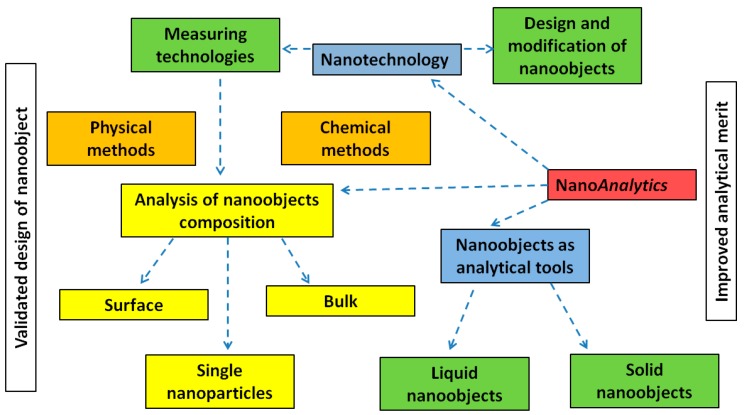
The proposed concept of nanoanalytics. Reproduced and adapted with permission [32]. Copyright Walter de Gruyter GmbH, Berlin/Boston, 2018.

The terms “nanoanalytics” or “nanoanalysis” do not have a common definition. However, recently it was summarized as follows—“Nanoanalytics is an area of analytical chemistry that develops the principals and methods of application of nanotechnologies and specific properties of nanoscaled objects in chemical analysis” [32]. At the same time, nanoanalytics consists of several branches: (i) application of various types of nanotechnologies in analytical chemistry; (ii) application of various nanoobjects as tools for chemical analysis; (iii) chemical analysis of nano-objects themselves via chemical and physical methods and corresponding metrological problems (see Figure 6). Below, we will consider the last approach used in nanoanalytics where nanoanalysis is accepted as a tool for nanoanalytics. Remarkably, for chemical or physical analysis at the nanoscale the utilized analytical assay must be extremely sensitive to visualize the minor changes in a nano-object’s architecture or chemistry (i.e., ppb, ppt level of concentration).

In general, two different conceptual strategies are distinguished in nanoanalysis [31,33]—the first is referred to as the methods for studying the morphology or exclusively elemental composition of nano-based samples, including scanning electron microscopy (SEM) coupled with energy dispersive X-ray microanalysis (EDX), transmission electron microscopy (TEM), glow discharge optical emission spectroscopy (GDOS), photo electron spectrometry (ESCA/XPS), optical microscopy (fluorescence, polarization, DIC). For quantitative determination of inorganic components, atomic absorption spectroscopy (AAS), flame emission spectroscopy and inductively coupled plasma atomic emission spectroscopy (ICP-AES) are widespread. It should be noted that the mentioned analytical methods aim to establish the size, shape, structure-property correlation, multidimensional characterization of the nanomaterials and their simple elemental analysis. More seldom, for nanoobjects characterization contact angle measurements and molecular composition methods, namely Fourier-transform infrared (FTIR) and RAMAN spectroscopy, Secondary ion mass spectrometry (TOF-SIMS) and Secondary Neutral Mass Spectrometry (SNMS) can be used. Remarkably, the characterization methods aim to describe the nano-based objects, to visualize them and sometimes to manipulate them (to analyze organic components). A trend has appeared in the application of the above mentioned methods for the characterization of nano-based objects and the quantification of individual elements on their surfaces [33].

**Figure 7:**
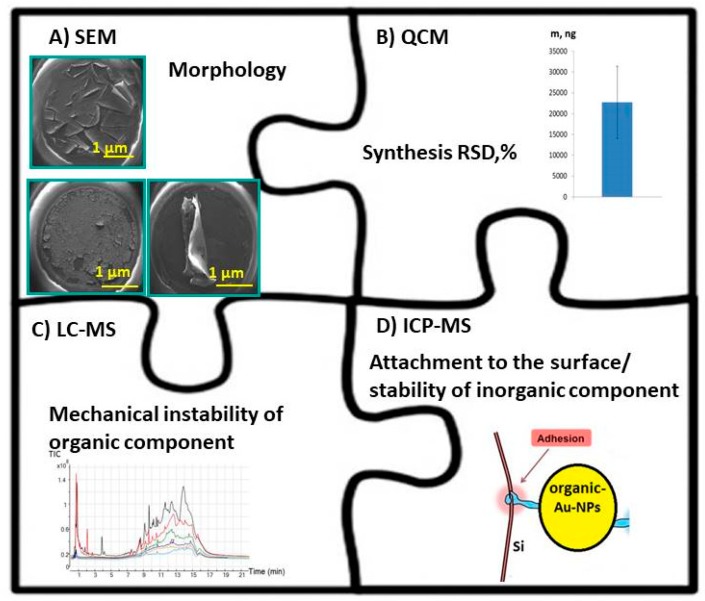
The summary of the several nanoanalytical assays used for the characterization, verification and validation of the design of the nanohybrids. (**A**)—SEM analysis; (**B**)—piezoquartz Crystal Microbalances (QCMs); (**C**)—liquid chromatography mass spectrometry (LC-MS) assay; (**D**)—inductively coupled plasma mass spectrometry (ICP-MS). The figures (B) and (C) were reproduced and adapted with permission [36,38]. Copyright Elsevier, 2018; Royal Chemical Society, 2019.

Another more seldom used strategy in nanoanalytics and nanoanalysis is to combine several functions in one methodology [31,33]. The second concept allows one to study the chemical composition of nano-based surfaces ideally combined with a study of their morphology in a comprehensive manner as summarized in Figure 7. Herein, the comprehensive global approach combining a tandem of the SEM technique for characterization of surface morphology, piezoquartz Crystal Microbalances (QCMs) for evaluation of the synthesis reproducibility, liquid chromatography mass spectrometry (LC-MS) assay to study the surface stability of organic component and ICP-MS to investigate the attachment phenomenon of organic-inorganic hybrids to the solid template is shown as example. Thus, SEM (see Figure 7A) provides the possibility of getting quick information about the impact of the certain experimental conditions on the design of the hybrid structures and the presence/absence of the possible morphological defects within the surface [34]. SEM with EDX is the best known and most widely used of the surface analytical techniques. High resolution images of surface topography, with excellent depth of field, are produced using a highly-focused, scanning electron beam which enters a surface with energy of about 0.5–30 kV resulting in the generation of many low energy secondary electrons. The intensity of these secondary electrons is largely governed by the surface topography of the nano-objects. The combination of SEM or TEM microscopy with EDX has been proven to be a powerful and relatively quick characterization tool for the morphological and chemical characterization of bulk and individual nanoparticles in various materials (inorganic component of O-I hybrids as a case study) [35]. SEM, accompanied by EDX analysis, is considered a relatively inexpensive and basically non-destructive approach to surface analysis. However, the preparation of samples can result in artifacts and a low ability to perform quantitative elemental analysis, which is considered a method limitation. In addition, SEMs are limited to solid samples small enough to fit inside the vacuum chamber that can handle moderate vacuum pressure. Moreover, SEM/EDX allows elemental characterization of the mostly inorganic content of the hybrid nano-objects [36,37,38,39].

To estimate the synthesis reproducibility of electroplated deposits, piezoquartz Crystal Microbalances (QCMs) are widely used (Figure 7B) [40,41]. In QCMs measurements, frequency shifts represent the weight of solid deposits with a high precision. For example, the reproducibility of organic-inorganic hybrids via co-deposition over 30 s, that is, α-cyano-4-hydroxycinnamic (CHCA) and α-cyano-2,3,4,5,6-pentafluorocinnamic acid (FCCA) together with Pd-NPs, was recently demonstrated [38]. The same nanoanalytical method can be utilized to study the adsorption phenomena of proteins, nanoparticles or polymer films to the different kind of solid surfaces in both static and dynamic mode or to investigate the adsorption kinetic during their attachment. Remarkably, QCM allows for the reproducibility values of the overall synthesis process of nanoobjects with nanogram precision [42]. On the other side, QCM is absolutely unable to provide analytical information about the chemical profile or species contributed nanohybrids.

The progress in mass spectrometry during the last decades has caused an increased impact on the characterization of micro- and nano-hybrid objects. Thus, the liquid chromatography mass spectrometry (LC-MS) assay in a droplet for characterization of the hybrid organic layers, for example, to study the polymer layer (Nafion©117) attachment to the biosensor surfaces was optimized (see Figure 7C) [36].

In was concluded that LC-MS after ligand cleavage is the only method for the qualitative and quantitative analysis of multiple functionalized hybrid nanostructures [43,44,45,46,47]. Zhou et al. illustrated that LC-MS is an important tool for identifying and quantifying individual ligands of multi-functionalized Au-NPs which were cleaved from the surface by reaction with iodine [47].

In contrast, to verify the stability or attachment of inorganic component of the hybrid nanomaterials to the solid template or to study the release of an inorganic mediator (e.g., Prussian Blue/Prussian White RedOx couple used for glucose monitoring at low potentials) from the biosensors, inductively coupled plasma mass spectrometry (ICP-MS) providing quantitative analysis at trace and ultratrace levels was successfully utilized (Figure 7D) [48,49,50]. However, ICP-MS requires sample preparation steps, viz. total and sequential extraction methods [51]. The purpose of sequential selective metal extraction is to mimic the release of the selective metals into solution under various environmental conditions. The leachate from the sequential extraction method must then be digested and analyzed on an inductively coupled plasma atomic emission spectrometer (ICP-OES) or an ICP-mass spectrometer (ICP-MS), depending on the element concentration level. Factors such as (a) pH of the acid used for adjustment, (b) temperature and (c) duration of extraction are the critical factors that need to be controlled. The optimized pre-treatment procedure should be reproducible, reliable and satisfy the requirement of followed quantitative analysis [52].

It should be noted that, regardless of the sample preparation procedure, the conventional sample preparation protocols utilized prior to the subsequent ICP-MS analysis are not sample friendly due to application of microwave digestion, leaching, sequential extraction methods and acidification. Therefore, such analytical analysis is commonly used for a limited number of samples.

In contrast, a strong potential of high resolution ICP-MS towards non-destructive control and validation of inorganic component of nanohybrids was recently demonstrated [37]. Nevertheless, the above mentioned assay does not exclude the sample preparation step. As a result, there is an urgent need to be able to characterize nanomaterials in the production halls in order to optimize the synthesis process on the basis of the results when required.

In the last years, besides conventional ICP-MS, single particle inductively coupled plasma mass spectrometry (sp-ICP-MS) has become a powerful nanoanalytical tool used for the screening, detection and characterization of hybrid nanoparticles in the solutions [53]. This method can easily provide researchers with information pertaining to the size, size distribution, particle number concentration and major elemental composition with minimized sample perturbation [49,51,54,55,56].

In comparison to the conventional ICP-MS or sp-ICP-MS performed in solutions, the laser ablation coupled ICP-MS (LA-ICP-MS) is one of the most exciting analytical technologies available due to the ability to perform ultra-highly sensitive chemical analysis down to ppb (parts per billion) levels without any extra sample preparation procedure [57,58,59]. Laser ablation is a process in which a laser beam is focused on a sample surface to remove the targeted material from the irradiated zone. Laser ablation has been considered and used for many technical applications, including the production and characterization of nano-objects, deposition of thin hybrid metallic and dielectric films, fabrication of superconducting materials and micromachining of nanostructures. LA-ICP-MS begins with a laser beam focused on the sample surface to generate fine particles. The ablated particles are then transported to the secondary excitation source of the ICP-MS instrument for digestion and ionization of the sampled mass. The excited ions in the plasma torch are subsequently introduced to a mass spectrometer detector for both elemental and isotopic analysis. The analytical merit of the approach appears in the advanced trace analysis, local inclusion and defect analysis, depth profiling, elemental and isotope mapping [60,61,62].

Remarkably, the investigation and analysis of nanometer-scale structures is having a major impact on the development of innovative nano-based materials. However, nanomaterials and nanocomposites are almost exclusively characterized through offline analysis methods at present. As a result, the characterization is separated from production in terms of time and space. Many of these analysis methods are very sensitive to fluctuations in environmental conditions (e.g., temperature, humidity, vibrations, etc.). Furthermore, characterized samples often require laborious preparation steps (e.g., thinning, grounding, etc.) and, in many cases, this is only possible for the lab scale experiments. In addition, the majority of characterization methods require a relatively long period of time to collect the measurement data as well as for data processing and so cannot be directly employed in the process.

In this regard, the usage of RAMAN or FTIR spectroscopy in situ providing molecular analysis (organic component of the nanohybrids) without the needs of addition sample preparation step can be considered a desirable alternative [63,64]. Thus, to monitor the surface chemistry of the hybrid nanostructures, FTIR emission spectra in transmission mode from the scratched solid hybrids can be used. Remarkably, FTIR provides molecular analysis (organic layer finger-prints, viz. polymer membrane or enzyme layer) versus elemental analysis recorded by EDX screening. In this case, typically polymer or enzyme characteristics lines and peaks intensities are used to control the quality of the hybrid co-deposits depending on the preparation conditions. RAMAN spectroscopy can be extremely helpful for the identification and quantification of chemical species as well as for chain orientation analysis in hybrid nanomaterials, characterization of multilayered systems, crystal size determination, crystallization degree estimation, stress, defects and contaminants verifications [65,66].

**Figure 8:**
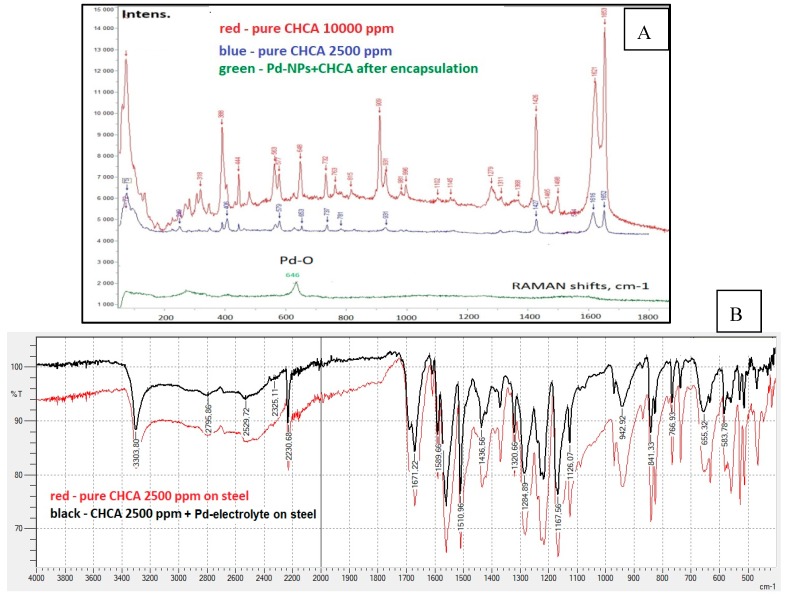
The RAMAN (**A**) and FTIR (**B**) transmission spectra obtained from the same O-I nanohybrids. O-I nanohybrids were prepared by the co-deposition approach from the Pd-electrolyte contenting α-cyano-4-hydroxycinnamic acid (CHCA). The preparation conditions of the O-I nanohybrids were reported in Reference [38]. All the analyses were conducted in the laboratory of the presented authors. Raman investigations were carried out under ambient conditions on a LabRAM HR Evolution HORIBA Jobin Yvon A Raman microscope (Longmujeau, France) using a 633 nm He-Ne laser (Melles Griot, IDEX Optics and Photonics, Albuquerque, NM, USA). FTIR measurements were conducted on an FT-IR spectrometer IRSpirit (Shimadzu, Tokyo). To control the instrument parameters and perform the data analysis, Lab Solutions IR software (Shimadzu, Tokyo) was used.

However, the mentioned chemical analytical methods are limited towards quantitative analysis (limit of detection of targeted elements is often given in % [67,68]) and they do not allow for the detection of minor changes in the architecture and chemistry (ppm, ppb, ppt) of the nanohybrids (see Figure 8A,B).

Thus, FTIR often does not allow observing the difference in the samples molecular chemical profile due to the strong interference coming from the substrate/carrier (see Figure 8B, substrate—steel) and therefore, cannot be implemented as a reliable nanoanalytical tool to visualize the small changes in the chemistry of the O-I nanohybrids.

For the characterization of nanohybrids with biological receptors, surface plasmon resonance (SPR) was proven as a reliable technique for studying interactions between biomolecules, that is, their binding characteristics, affinity degree and biochemical mechanisms [69,70,71,72].

SPR is based on an optical phenomenon that enables monitoring of changes in refractive index via a quantum mechanical principle. Remarkably, the optical measurement system detects changes occurring on the sensor surface [69,70,71,72].

The great advantage of SPR for bio-based nanohybrids is a visual representation of the molecular interactions in a time-dependent manner in a form of the simple sensorgrams. Thus, based on the sensorgram it is readily possible to obtain the key information on kinetics involved. Moreover, SPR allows to conduct the experiment under the physiologically relevant conditions that is important for life-science, electrochemistry, point of care (POC) technologies, food and environmental safety.

The potential limitations of the SPR include the non-specific bindings to the outcome signals, possible change in ligand structure in comparison to its native configuration upon immobilization on the sensor surface that significantly affects the sensitivity, selectivity, resolution and the limit of detection of the method [69,73]. Moreover, the kinetic rates data for biomolecular species obtained by SPR have low significance and analytical merit in the hybrid POC biosensor systems.

Importantly, for characterization of polymer contenting nanohybrids, time-of-flight secondary ion mass spectrometry (TOF-SIMS) has been proven as a powerful tool for nanoanalysis due to a high method sensitivity, high dynamic range, specificity and selectivity. TOF-SIMS can provide information about oligomer distributions, average molecular weights, fingerprint patterns for polymer identification, monomeric unit sequences, branching, cross-linking substitution, copolymer structures and additives or impurities [74].

**Figure 9:**
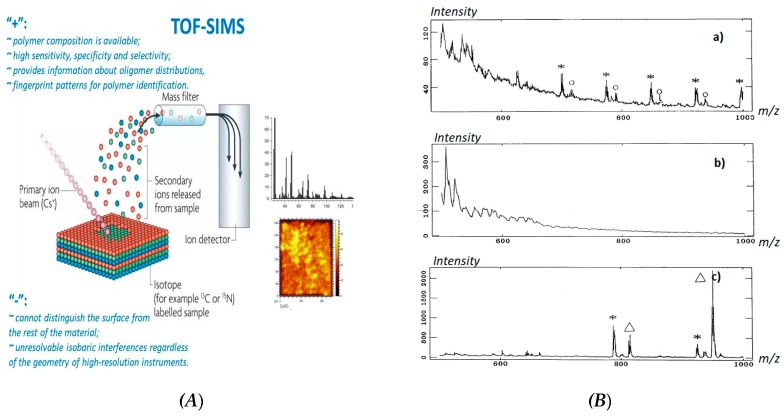
The simplified operating principal of time-of-flight secondary ion mass spectrometry (TOF-SIMS) as a tool for trace chemical analysis of polymer-based hybrids (***A***) and typical TOF-SIMS low mass range spectra (***B***) obtained for polysiloxanes-silver hybrids: (**a**) polydimethylsiloxanes (PDMS), (**b**) polyhydromethylsiloxane (PHMS), (**c**) polymethylphenylsiloxane (PMPhS). Note: (asterisk) [*nR*+Ag]^+^, (circle) [*nR*+16+Ag]^+^, (triangle) [*nR*+162+Ag]^+^ ([oligomer+Ag]^+^). Note: the sum of the molecular weights of the two end groups, H and CH_3_, is 16; the sum of Me_3_Si and OSiMe_3_, is 162 [78]. Reproduced and adapted with permission [78]. Copyright Springer Nature, 1998.

However, major challenges, i.e., distinguishing the surface from the rest of the materials, isobaric interferences regardless of the geometry of high-resolution instruments still remain unsolved (Figure 9A) [75,76].

Moreover, TOF-SIMS as a characterization method is very time consuming and requires a highly skilled person with a strong background in conventional and nanoanalytical chemistry, broad knowledge in sample preparation techniques and data interpretation methods [77]. For example, to distinguish the difference within the polymer networks, cationization by silver ions might be requested. In this case, the siloxane samples must be pretreated in toluene followed by deposition onto silver targets [78]. Afterwards, the silver substrates must be etched in nitric acid and ultrasonicated in distilled water for about 3 min, then rinsed in distilled water and methanol. As a result, the obtained mass spectra can be attributed to the O-I hybrid material (polymer-silver) and three polydimethylsiloxanes (PDMS), polyhydromethylsiloxane (PHMS) and polymethylphenylsiloxane (PMPhS) and the effect of functional groups can be distinguished by the changes of polymer fragmentation mechanisms (Figure 9B).

The manufacturing of the novel nano-objects, i.e., O-I nanohybrids can benefit from the usage of the direct and easy-to use nanoanalytical tools and approaches by direct detection of the surface chemistry of intact samples, their impurity degree and synthesis defects. The usage of such crucial and reliable tools or optimized methodologies will help to improve the quality and reliability of nano-based end-products.

## 4. Nanoanalytics on the Template: LDI-MS as a Crucial Tool towards Nanohybrids Characterization

As an alternative to LC-MS and TOF-SIMS, matrix-assisted laser desorption ionization mass spectrometry (MALDI-MS) was reported as a reliable analytical tool towards characterization of O-I nanohybrids [79]. Thus, Yan et al. used MALDI-MS to get a semi-quantitative measure of the ligand composition of mixed-monolayer Au-NPs by detection of the individual ligands. However, the application of MALDI-MS for the analysis of small molecules (<700 Da) still remains a great challenge due to the interference from the matrices in the low mass region on the one hand and the presence of the “sweet spot” phenomenon on the other hand [80,81].

Therefore, in the last two decades, more attention was paid to the MALDI-free methods in analytics. Thus, the progress in nanotechnology caused the appearance of the novel analytical tools, that is, nanomaterials-assisted laser desorption/ionization or surface-assisted laser desorption/ionization mass spectrometry (often abbreviated as LDI-MS) utilizing nanomaterials as a template instead of the organic matrices. In LDI-MS platforms, the role of hybrid nanosubstrates is to provide a homogeneous analyte distribution over the nanostructured surfaces and to guarantee the effective analyte desorption. Moreover, in LDI-MS, the surface of the nano-object is involved into the transfer of proton to the bioanalyte. Alternatively, the nanostructured surface can be a donor of other reagent ions (K^+^, Na^+^, Me^n+^, etc.), which are essential to provide the effective ionization of the targeted bioanalyte [82]. Recent advances in nanomaterials for LDI-MS analysis of small molecules were summarized by Shi et al. [83].

It should be noted that, in the majority of cases, LDI-MS related studies rely on the similar protocols including (i) a fabrication step of the novel nanobased substrates, (ii) characterization of the nano-templates followed by (iii) subsequent analysis of the targeted bioanalyte by means of MS and (iv) comparison of the obtained analytical merit. Sadly, the current state-of-art in the development of LDI-MS follows a largely empirical approach that can be summarized as—*“if it works it is good.”* Nevertheless, to stimulate the progress in the LDI-MS with enhanced analytical merit, a better understanding of the operating principles and key aspects affecting the system response are necessary.

Our research group has invested several years to understand the impact of certain parameters, including surface melting/sintering effect, thermoconductivity, availability of reagent ions, ablation rates, surface acidity/basicity and crystallization properties of the analyte on the LDI-MS signal [82,84,85]. Remarkably, it was noted that even small changes in the nanosubstrate morphology, its architecture and chemistry can dramatically affect the quality of the obtained LDI-MS response even for the same targeted analyte. Thus, from the one LDI target to another target produced in accordance to the same protocol, the type of generated analyte species might be presented as M+H^+^, M+K^+^, M+Me^n+^ and so forth. Significantly, an intensity of analyte fragmentation, a type of observed fragment and obtained S/N ratio can be very different. It is believed that this phenomenon can be explained in terms of poor synthesis reproducibility from one LDI target to another, from batch-to-batch resulting in the different inter- and intra-day target behavior and a different LDI signal even for the same type of the targeted bioanalyte. In fact, the minor changes in the nanomaterial (target) design and fabrication process resulted in the random overall LDI response.

Initially driving by the idea to develop a universal LDI-MS target based on O-I nanohybrid materials and by the attempts to understand the key parameters affecting the LDI-MS system response, we came to the statement that this platform can be used for the reliable characterization of the O-I nanohybrids (organic- or bio-component). Thus, LDI-MS can be readily applied as a crucial, sensitive and reliable nanoanalytical tool for monitoring of the quality of the produced O-I nanohybrids, estimation of the synthesis efficiencies of the used protocols, reproducibility of the synthesis, screening the deposition rates of the organic component in the complex nanohybrids, verification of the surface chemistry and purity/impurity degrees of the nano-substrates and/or understanding of the degradation mechanism of the nanohybrids.

Herein, we do not aim to demonstrate LDI-MS again as a platform for the specific analysis of biomolecules which is more common in analytical chemistry and mass spectrometry but we highlight the potential of this method as a nanoanalytical tool for analysis and characterization of the hybrid nano-objects. In other words, we demonstrate the potential of LDI-MS in nanoanalytics in the following manner—a big step from the routine analysis of small molecular weight organic compounds to a crucial platform for monitoring the quality of the nano-based hybrid materials.

**Figure 10:**
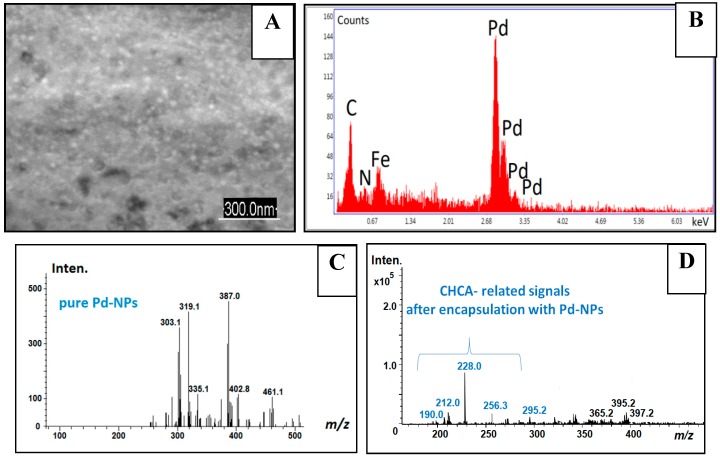
The scanning electron microscopy (SEM) images (**A**), Energy dispersive X-ray (EDX) spectra (**B**) and laser desorption ionization (LDI-MS) spectra (**C**,**D**) obtained from the same organic-inorganic nanohybrid prepared by the co-deposition approach from the Pd-electrolyte supplemented by α-cyano-4-hydroxycinnamic acid (CHCA), 189.17 g/mol. The preparation conditions of the O-I nanohybrids and experimental parameters were reported in Reference [38]. Reproduced and adapted with permission of [38]. Copyright Royal Chemical Society, 2019.

Thus, LDI-MS showed a great promise for fine tuning of the design and the performance of the hybrids for further implementation in their advanced nanomaterials, sensing applications, point of care medical microdevices, biotechnology and biochip development (see Figure 10). Remarkably, by means of SEM/EDX analysis (Figure 10A,B) typically used for hybrid nano-objects characterization, it was possible to exclusively verify the qualitative presence of Pd-line, (possible related to Pd-NPs deposited onto the steel template) [38]. To approve the successful encapsulation of organic content (α-cyano-4-hydroxycinnamic acid, CHCA), the method capacity was not enough. Thus, the C-and N-lines visible on the EDX spectra are not reliable markers for CHCA presence and can be attributed to the sample’s environment. Also, it was not possible to prove the encapsulation of CHCA by means of RAMAN or FTIR studies (see Figure 8). In contrast to performed SEM/EDX, RAMAN or FTIR analysis, the study carried out according to LDI-MS algorithm (nano-object → laser beam → mass spectra → chemical finger-print image) revealed the clear presence of CHCA after performed encapsulation (Figure 10C,D) [38]. Once the laser beam was applied to the hybrid targets, the CHCA related signals were seen in a positive detection mode at *m/z* 190, 212.0 and 228.0 that corresponds to [CHCA+H]^+^, [CHCA+Na]^+^ and [CHCA+K]^+^ adducts, respectively.

Another example to illustrate the potential of the platform could be the verification of Triton X-100 presence onto the nanostructured surface. Triton X-100—is a widely used polymer in O-I hybrids and biosensors development that can be deposited onto the electrode surface by means of different strategies, including drop-casting method or by electropolymerization [86,87].

**Figure 11:**
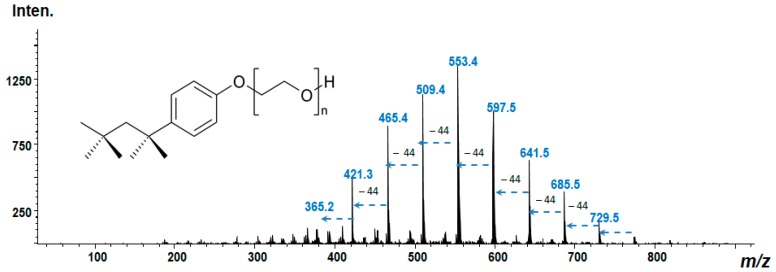
Mass spectrum obtained from the electroplated Ag-NPs (electrolyte composition see above) deposited with Triton X-100 (500 ppm in solution) during 1 min at the cathodic current of 20 mA. Note: exclusively polymer-corresponding fragments are observed on the spectra. All the analyses were conducted in the laboratory of the presented authors. The experimental parameters were reported in References [82,84,85].

In our laboratory, we performed a simple co-deposition of Triton X-100 with Ag-NPs from polyelectrolyte solution (AgCl, 3 g/L; K_4_[Fe(CN)_6_], 10 g/L; Na_2_CO_3_, 25 g/L) followed by the subsequent analysis in the manner as described above. Thus, the produced nanohybrid was adapted onto the conventional MALDI plate. The mass spectrum obtained in a positive detection mode is presented in Figure 11. Remarkably, despite the simplicity of the used deposition procedure, the repeatable characteristic finger-prints fragments (–[C_2_H_4_O]-units) corresponding to the Triton X-100 were clear visible on the mass spectra. In fact, Triton X-100 is a mixture of ethoxylated octylphenols. These compounds differ by 44 ([C_2_H_4_O]-units) atomic mass units according to their degree of ethoxylation [88].

In this particular case, LDI-MS was applied to verify the co-deposition/co-encapsulation of polymer layer together with Ag-NPs onto the template under the used experimental conditions.

## 5. LDI-MS as a Useful Tool in Biosensors Design Screening, Standardization and Validation

Besides the verification of the small molecular weight organic compounds as a part of the nano-hybrids in the manner as appended above, we do believe that the LDI-MS nanoanalytical platform has strong potential to be implemented as a crucial tool for the monitoring of fabrication processes and design screening/optimization in nanobiosensors (i.e., where the biosensors design involves the encapsulation of bioreceptor, including enzyme, antibody and so forth, and polymeric membranes) [89,90].

**Figure 12:**
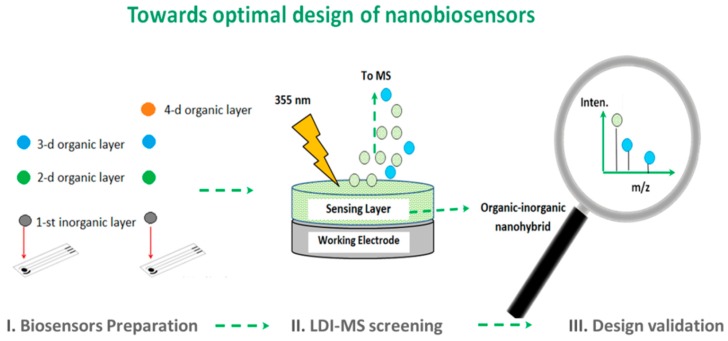
Schematic workflow for LDI-MS applied for characterization of the hybrid nanobiosensors towards design optimization.

It is interesting that, in order to improve the overall biosensors performance, i.e., sensitivity, selectivity, limits and speed of analyte detection and so forth, a great majority of novel nanomaterials were readily implemented in this field [91]. At the same time, due to the complexity of the biosensing mechanisms or lack of required multi-analytical techniques or skills, the impact of preparation conditions on the design of nanohybrids and their analytical response is mainly characterized in the presence of analyte during electrochemical studies (e.g., chronoamperometry, cyclic voltammetry, etc.). However, in reality, such an approach cannot be informative enough to provide the researcher with a correct understanding and correlations between the quality of integrated nano-structures or objects and their influence on the biosensor response [89,90]. In this case, the LDI-MS sensing platform, by revealing the exact mass of organic compound (enzyme or polymeric membrane) or its environmentally stable fragments, makes the screening of the hybrid nanobiosensors possible and allows to obtain important information on the biosensors synthesis and operational stability. The schematic workflow for LDI-MS applied for characterization of the nanobiosensors is summarized in Figure 12.

It is also important to point out that already at the biosensor preparation step (Figure 12) both physical and chemical factors strongly effect the final composition of the nanobiosensor, which means that initial composition of the materials and/or solutions are for sure not going to represent the final NPs structure, bioreceptor conversion, ligands ratio and so forth. Thus, there is clear evidence of speculative nature of current scientific understanding of biosensing response that is related to the surface modifications via multi-functionalized NPs and biologically active components when the structured analytical characterization strategy of such NPs is missing [47].

Therefore, LDI-MS analysis represents a nice alternative for characterization of nano-objects (except pure metal nanoparticles based or pure semiconductor surfaces) and simultaneous screening of the surface modifications together with the identification of the impurities level below 0.1% ppm. For a comparison, the alternative semi-quantitative EDX or RAMAN spectroscopy analysis is only able to provide reliable data for surface chemistry functionalization and/or characterization at the percentage concentration levels [35,67,68].

**Figure 13:**
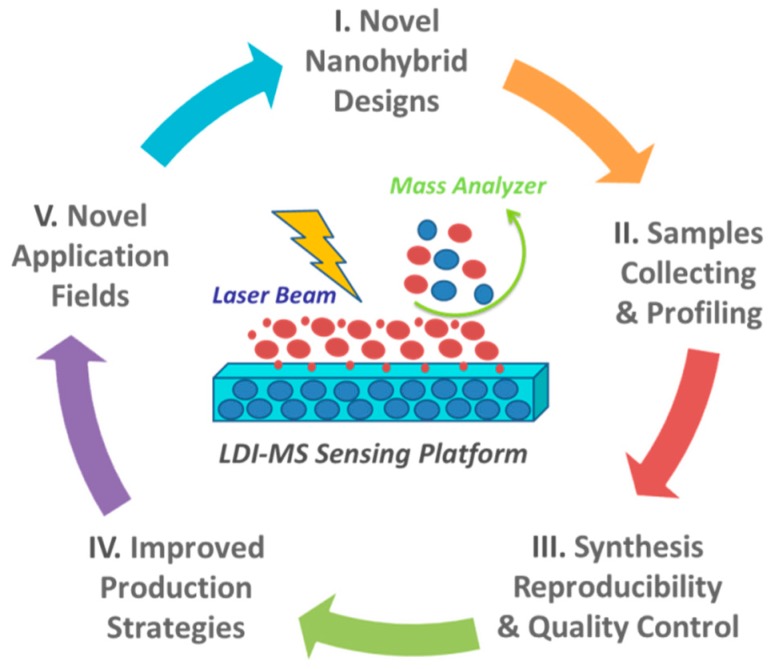
Schematic workflow and score of applications of LDI-MS analysis as a crucial nanoanalytical tool for nanohybrids development.

The score of applications of LDI-MS as a nano-analytical tool is presented in Figure 13.

Thus, by means of an LDI-MS sensing platform, novel nanohybrid-based designs can be characterized versus the stability of produced nano-objects and structures. It allows the fast identification of the unstable system parameters and helps with further quantification of the synthesis and composition reproducibility either during manufacturing or application steps. In other words, such a concept offers an alternative towards quality control of the produced nanohybrids.

Furthermore, a better understanding of fundamental principles behind the nanohybrids preparation step leads not only to the improvement of the production strategy itself but reveals the ideas for the nanohybrid design adjustment that subsequently opens up the possibilities for their novel applications (Figure 13).

Moreover, it is expected that LDI-MS can be utilized as a direct and reliable tool to study the mechanism of biocatalytic reaction [92]. Thus, recently, Hung and co-authors explored the potential of the LDI-MS platform towards the investigation of the catalytic mechanism of the core-shell NH_2_-organosilica structured nanoreactors with Pt-NPs for the detection of metabolic biomarkers. Therefore, LDI-MS can be readily helpful in the future for the identification of the key reactants and products in nanoreactors that can gain to elucidate diverse biocatalytic reactions.

In addition, LDI-MS could significantly contribute to our understanding of underlying degradation mechanisms of organic component within O-I nanohybrids depending on the environmental, operational and storage conditions (for example, photocatalytic degradation of the azo dyes, enzymes/co-factors or polymer membranes onto nanotemplates or biosensors) [93]. This knowledge would help us to develop the protection strategies towards the long-term stability of O-I nanohybrids.

**Figure 14:**
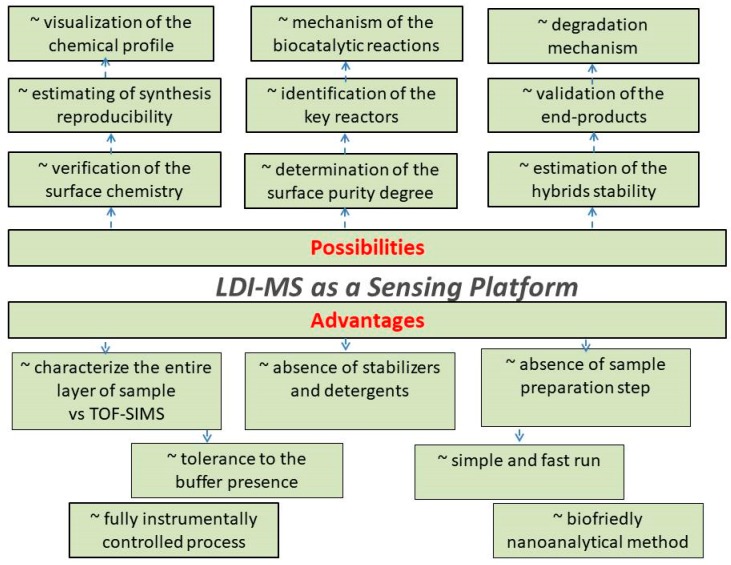
The proposed concept of LDI-MS as a sensing/quality platform for characterization of the O-I hybrid nanomaterials.

To summarize, the manufacturing of the novel nano-objects, including O-I nanohybrids can also benefit from the usage of correctly implemented tools and nanoanalytical approaches by detecting surface chemistry, impurity degree, synthesis defects, thus improving the quality and reliability of end-products. Ideally, these nanoanalytical techniques or tools applied for the characterization of the O-I hybrid nanomaterials should satisfy multi-criteria—(i) provide the information about the surface chemistry in a condensed and in a clear form; (ii) deliver the information an average molecular weights, fingerprint patterns identification for organic component; (iii) provide the information about presence of additives or impurities; (***iv***) to illustrate the intensity of possible fragmentation degrees, Figure 14.

Furthermore, based on results obtained via LDI-MS (surface purity or impurity degree, S/N ratio, typical adducts or fragments, etc.), the general criteria and guidelines for quality tests and comprehensive characterization of end-nanomaterials can be developed, which would be the next step towards standardization of the nanoobjects.

## 6. Future Perspectives

The optimization, validation and use of nanoanalytical tools, assays and strategies for the characterization of nano-objects in general, and O-I hybrids as a case study, represent a set of developments in which specificities are based on the singularities of its targets. At the crossroads of physics, chemistry, nanoscience and biochemistry, the nanoanalytics highlight the remaining needs in modern hybrid nanomaterials and nanobiosensors. Depending on the general interest (study of nanomaterials stability, leakage issue, dissolution, agglomeration/aggregation, synthesis process reproducibility from batch-to-batch or responses/signals reproducibility obtained from the nanohybrids, their design optimization, identification of the optimal operating conditions, study of hybrids deposition rates, verification of the surface chemistry or purity/impurity degree, etc.) certain evaluation criteria should be developed.

Obviously, there is still much to be done in terms of methodology, including the development of the general nano-characterization platform and metrological guidelines for the nanohybrid´s design and validation. For example, the formation of the universal decision making platform towards the usage of a certain nanoanalytical tool depending on the type of hybrid nanomaterial, its dimensionality, required resolution and criteria towards validation would be a desirable alternative.

Furthermore, without methods to characterize and quantify the O-I nanohybrids, the percentage of each ligand on the NPs/inorganic component can only be speculated upon on the basis of the material ratio used in the reaction. However, due to the competition of reactions and solubility difference, the ratio of starting materials does not reflect the final ligand ratio in the multi-functionalized final O-I nanomaterial. Anyway, it is clear that none of the existing analytical methodologies or tools can be used as a universal one for the general characterization and quantification of O-I nanohybrids.

In this regard, following the results demonstrated by way of LDI-MS, the general workflow towards final nano-based products design and quality characterization can be proposed, which in principle can become a crucial platform for the screening and standardization of O-I nanohybrids.

## 7. Conclusions

Overall, versatile types of O-I nanohybrids can offer an advanced platform for the future development of point-of-care technologies, bio-and environmental sensing, lab-on-chip technology and personalized medicine based on innovative and highly reproducible nanomaterials, recyclable, self-assembling, self-repairing or even self-replicating with an advanced analytical merit. However, the progress in this research field is not possible without the development of reliable nanoanalytical tools and methodologies for the standardization and validation of the novel nanohybrids.

Importantly, the nanoanalytical techniques mentioned in this review require a fundamental understanding of the physical processes between nano-objects (O-I nanohybrids as a case study) and detection techniques (tip (AFM), electron (SEM/EDX, SIMS) or laser beam (LA-ICP-MS, LDI-MS, RAMAN spectroscopy, FTIR, etc.), which can be investigated by simulations or modeling approaches to gain a deeper understanding of processes and reliable physical models. The developments in this field will help the extraction of a maximum of analytical information from the single run or datasets. To address this, the developments in both nanoanalysis tools (instrumentation), as well as new nanoanalytical methodologies, are expected.

Herein, the potential of LDI-MS as a crucial nanoanalytical platform and tool for nanoanalysis utilized for the monitoring of synthesis reproducibility, surface chemistry and the quality of O-I hybrid nanomaterials was highlighted. We believe this analytical platform will, in the future, fill one the most important niches for efficient nanohybrids’ characterization, standardization and validation.

It should be noted that the typical “hybrid scientists” can make bridges between different research fields including mineralogy, biology, organic and inorganic chemistry, polymer science, material engineering, nanoscience and physics. Obviously, to avoid situations in which excellent scientific results might be readily transformed into useless information, the list mentioned above should be supplemented by nanoanalytics. Nanoanalytics can transform the current scientific “failures” into tomorrow’s successes.”
